# GIPC1 promotes tumor growth and migration in gastric cancer via activating PDGFR/PI3K/AKT signaling

**DOI:** 10.32604/or.2023.043807

**Published:** 2023-12-28

**Authors:** TINGTING LI, WEI ZHONG, LIU YANG, ZHIYU ZHAO, LI WANG, CONG LIU, WANYUN LI, HAIYAN LV, SHENGYU WANG, JIANGHUA YAN, TING WU, GANG SONG, FANGHONG LUO

**Affiliations:** 1 Cancer Research Center, School of Medicine, Xiamen University, Xiamen, 361000, China; 2 Department of Pharmacy, Xiamen Mental Health Center, Xiamen Xianyue Hospital, Xiamen, 361000, China

**Keywords:** GIPC1, PDGFR, Gastric cancer, Proliferation, Migration

## Abstract

The high mortality rate associated with gastric cancer (GC) has resulted in an urgent need to identify novel therapeutic targets for GC. This study aimed to investigate whether GAIP interacting protein, C terminus 1 (GIPC1) represents a therapeutic target and its regulating mechanism in GC. GIPC1 expression was elevated in GC tissues, liver metastasis tissues, and lymph node metastases. GIPC1 knockdown or GIPC1 blocking peptide blocked the platelet-derived growth factor receptor (PDGFR)/PI3K/AKT signaling pathway, and inhibited the proliferation and migration of GC cells. Conversely, GIPC1 overexpression markedly activated the PDGFR/PI3K/AKT signaling pathway, and promoted GC cell proliferation and migration. Furthermore, platelet-derived growth factor subunit BB (PDGF-BB) cytokines and the AKT inhibitor attenuated the effect of differential GIPC1 expression. Moreover, GIPC1 silencing decreased tumor growth and migration in BALB/c nude mice, while GIPC1 overexpression had contrasting effects. Taken together, our findings suggest that GIPC1 functions as an oncogene in GC and plays a central role in regulating cell proliferation and migration via the PDGFR/PI3K/AKT signaling pathway.

## Introduction

Gastric cancer (GC) is one of the most common malignancies and the fourth-leading cause of cancer mortality worldwide [1]. The development and progression of GC are affected by multiple factors, such as family heredity, diet, alcohol consumption, smoking and *Helicobacter pylori* infections [1,2]. The prognosis of GC is poor as the disease is first diagnosed at an advanced stages. Although there are numerous treatment strategies for GC, surgery is currently the most effective treatment with a 5-year survival rate of only 20% [3]. The molecular mechanism of GC development and progression needs to be elucidated urgently, owing to the high morbidity, high mortality, and complicated pathogenesis associated with the disease.

GIPC1, C-terminus of GAIP interacting protein, also known as synectin, is a member of the PDZ domain protein family. It functions as an essential trafficking adaptor for membrane receptors, signaling effectors and protein complexes [4]. GIPC1 consists of the GH1, PDZ and GH2 domains. The GH1 domain within the N-terminal region of GIPC1 participates in dimerization and the C-terminal region of GIPC1 (GH2 domain) interacts with myosin VI [5,6]. The PDZ domain of GIPC1 interacts with and stabilizes numerous transmembrane proteins [insulin like growth factor 1 receptor (IGF-1R), neurotrophic receptor tyrosine kinase 1 (NTRK1), adrenoceptor-β1 (ADRB1), dopamine receptor D2 (DRD2), transforming growth factor β receptor 3 (TGFβR3), LDL receptor-related protein 1(LRP1), neuropilin 1 (NRP1), glucose transporter type 1 (GLUT1), syndecan 4 (SDC4), semaphorin 4C (SEMA4C), integrin α5 and Van Gogh-like 2 (VANGL2)], cytosolic signaling regulating proteins, and viral proteins [7]. Thus, GIPC1 functions as an adaptor for loading PDZ-binding proteins as cargo onto the myosin VI motor protein, which transports various transmembrane proteins into endocytic vesicles [4,8] involved in processes such as endocytosis [6,7], angiogenesis [9], lymphangiogenesis [10], cell cycle regulation [11], and cancer metastasis [12]. GIPC1 is expected to become a novel and effective anti-cancer target. Elevated GIPC1 expression levels have been reported in several cancer types, including breast cancer [13] and pancreatic ductal adenocarcinoma [14,15], and have been revealed to be associated with a poor prognosis. GIPC deficiency inhibits proliferation and migration, and promotes apoptosis in numerous cancer cell types [11,16,17]. However, to our knowledge, our study is the first to elucidate the functions and mechanisms of GIPC1 in GC.

GIPC1 is a versatile adaptor protein that regulates the functional trafficking of receptor tyrosine kinases (RTKs), G protein-coupled receptors, TGFβ receptors and integrins [4]. PDGFR family proteins, including PDGFR-α and PDGFR-β, are classical proto-oncogenes that encode RTKs responding to the platelet-derived growth factor (PDGF) [18]. PDGFR promotes angiogenesis [19,20], lymphatic vessel formation [21], proliferation, and migration [22–24]. PDGFR is reported to be an important cancer promoter and potential therapeutic target for several human cancers, including breast cancer [21], pancreatic cancer [25], ovarian cancer [26], and GC [27]. Our previous study demonstrated that GIPC1 may be involved in liver fibrosis through the regulation of PDGFR expression [28]. GIPC1 promotes PDGF-dependent hepatic stellate cell activation by diverting of PDGFR-α from the selective autophagy pathway and through the epigenetic regulation of PDGFR-β. These findings form the basis of the present study, which aimed to investigate the effects of GIPC1 on PDGFR signaling and cellular functions in GC.

Our findings revealed that GIPC1 expression was elevated in The Cancer Genome Atlas (TCGA) database and GC tissues. Upon performing cell assay analyses, and transcript and protein analyses of tissues obtained from patients with GC and cell lines, our findings indicated that GIPC1 increased PDGFR-α and PDGFR-β expression levels. Thus, GIPC1 activated the PI3K/AKT signaling pathway and promoted the proliferation and migration of gastric carcinoma cells. The present study provides insight into the possibility that GIPC1 can be used effectively as a target for the development of therapeutic approaches for GC treatment in the future.

## Materials and Methods

### Reagents

MK-2206 2HCl was purchased from Selleck Chemicals (Houston, USA). GIPC1 and PDGFR-α and PDGFR-β antibodies were obtained from Proteintech Group, Inc. (Chicago, USA). The phospho-AKT (Ser473) antibody and phospho-PI3 Kinase p85 (Tyr458)/p55 (Tyr199) were purchased from Cell Signaling Technology, Inc. (Danvers, USA). The PI3Kinase p85α antibody was obtained from Abcam (Cambridge, UK).

### Cell lines and cell culture

Human GC cell lines HGC-27 (RRID: CVCL_1279); BGC-823 (RRID: CVCL_3360), and AGS (RRID: CVCL_0139) were obtained from the Cancer Research Center at Xiamen University (Xiamen, China). Cells were cultured in Dulbecco’s modified eagle medium (Gibco, Shanghai, China) containing 10% fetal bovine serum (FBS) (Gibco, Shanghai, China) at 37°C in an atmosphere with 5% CO_2_ and 95% humidity. In the past three years, all the cell lines were authenticated via short tandem repeat (STR) profiling. Briefly, genomic DNA was first extracted from the cell line, and primers for STR sequencing were designed, followed by PCR amplification and electrophoresis detection. Finally, the results of STR typing were compared with data from the STR database. These cell lines were confirmed to be mycoplasma-negative via a PCR-based method.

### Immunohistochemical analysis

Patient tissue microarrays were purchased from Shanghai Outdo Biotech Co., Ltd. (Shanghai, China) and included 80 normal gastric and GC tissues. The ethical review of this study protocol was approved by the Ethics Committee of Xiamen University and Shanghai Outdo Biotech Co., Ltd. All the patients signed informed consent forms. Tissue microarrays were deparaffinized and rehydrated. Subsequently, heat-induced antigen retrieval was performed, and sections were incubated overnight with specific antibodies at 4°C. After washing, the sections were incubated with a secondary antibody at 37°C for 1 h. The target protein in the sections was stained with 3,3′-diaminobenzidine, and the nuclei were stained with hematoxylin. Immunoreactivity was independently assessed by two pathologists who were blinded to the patient prognosis. If the differences between the results obtained by the two pathologists were comparatively significant, a third person would assess it. The intensity and area of staining were evaluated. The GIPC1 staining intensity was classified into the following four levels: 0, negative; 1, weak; 2, moderate; and 3, strong. The area of staining was based on the percentage of positively stained cells and was divided into the following five levels: 0, 0%; 1, 1%–25%; 2, 26%–50%; 3, 51%–75%; and 4, 76%–100%. The final values, obtained by multiplying the staining intensity and staining extent scores, were classified into the low (0–7) and high (8–12) groups.

### Vector construction and cell transfections

Short hairpin RNA (shRNA/sh) primers were designed based on the pLV-RNAi system, and the sequences used were as follows: GIPC1#1, GACATGATCAGCCAGCGTTCA; GIPC1#2, GCCATTGAGAAGGTGGATGAC; GIPC1#3, GCCATTGAGAAGGTGGATGAC; Flag-GIPC-forward, CCGCTGGGACTGGGGCGGC; and Flag-GIPC-reverse, CTAGTAGCGGCCGACCAAG.

The pLV-GIPC1-Puromycin or pLV-control-Puromycin lentiviral vector was included in the lentivirus together with the pMDLg/pRRE, pVSV-G, and pRSC-Rev plasmids. Subsequently, the packaged lentivirus was transfected into 293T cells. This lentivirus was used to infect BGC-823 and HGC-27 GC cells. Stably transfected cells were screened using puromycin in accordance with screening protocols.

### Quantitative PCR

The total RNA was extracted from cultured cells according to the protocal provided with the TRIzol reagent (Takara, Dalian, China), and RNA was reverse-transcribed into cDNA. Quantitative PCR analysis was performed using GAPDH as an internal control. The primer pairs used were as follows: GIPC1 forward, 5′-GCTGGAGAGTTACATGGGTATC-3′ and GIPC1 reverse, 5′-TCAGGGAAGGCAAAGTCAC-3′; PDGFR-α forward, 5′-GCGGCCGCATGGGGACTTCCCATCC-3′ and PDGFR-α reverse, 5′-CTCGAGCAGGAAGCTGTCTTCCACC-3′; PDGFR-β forward, 5′-AGTGATGTCTGGTCTTTTGGG-3′ and PDGFR-β reverse, 5′-TGGCATTGTAGAACTGGTCG-3′; and GAPDH forward, 5′-TTGGTATCGTGGAAGGACTC-3′ and GAPDH reverse, 5′-GACCTTGCCCACAGCCTTG-3′.

### Western blot analysis

Total protein was isolated using radioimmunoprecipitation (RIPA) lysis buffer (Beyotime, Shanghai, China) supplemented with phenylmethanesulfonyl fluoride (PMSF) (Beyotime, Shanghai, China). Sodium dodecyl sulfate—polyacrylamide gel electrophoresis (SDS-PAGE) was used to separate proteins with the same molecular weight. Proteins loaded in equal amounts were resolved using 10% SDS-PAGE and transferred onto polyvinylidene fluoride (PVDF) membranes. Membranes were blocked with 5% fat-free milk in Tris buffered saline with Tween 20 at room temperature for 1 h and incubated overnight with primary antibodies at 4°C. This was followed by incubation with a secondary antibody. Finally, proteins were detected using enhanced chemiluminescence (ECL) assays (Beyotime, Shanghai, China).

### Cell proliferation assay

Cell proliferation was determined using a Cell Counting Kit-8 (CCK-8) assay (Beyotime, Shanghai, China). Cells (3,000 per well) were seeded into 96-well plates along with 100 μL medium. PDGF-BB (PeproTech, State of New Jersey, USA) was added to the medium everyday at a final concentration of 80 ng/mL. The concentration of AKT inhibitor (Selleck, Houston, USA) was 2 µmol/mL. The concentration of PSQSSSEA was 80 μg/mL. Subsequently, the CCK-8 reagent (10 μL) was added to generate a working solution. After incubating the cells for 2 h, optical density values were read at 450 nm. Migration rate = (original blank area − 24 or 48 h blank area)/original blank area × 100%

### Wound healing experiment

A straight line was drawn on the back of the six-well plate, an appropriate number of cells was inoculated, and the plate was covered overnight. A straight scratch was made using a 200-μL sterile yellow tip to create a wound after treatment with 10 µg/mL mitomycin C (Selleck, Houston, USA) for 2 h. The plate was washed three times with phosphate buffer saline (PBS), and a serum-free medium was added. Images were captured under a microscope at 0, 24, and 48 h after scratching, and the data were analyzed using ImageJ software (National Institutes of Health, Bethesda, MD, USA).

### Transwell invasion assay

The invasion assay was performed using a 24-well plate transwell chamber containing 8-μm wells (Corning, USA). The top chamber contained Matrigel (BD Biosciences). Cells (1 × 10^5^/well) were inoculated in a serum-free medium in the top chamber, while the bottom chamber contained the medium, along with 10% FBS. The invasion of cells was allowed to occur for 48 h at 37°C, after which they were fixed with 4% paraformaldehyde and stained with 0.1% crystal violet solution. Photographs were obtained using cellSens Dimension.

### Immunofluorescence assays

Cells were incubated on coverslips and fixed with 4% paraformaldehyde solution for 20 min. Subsequently, cells were incubated with 0.2% Triton X-100 in PBS for 10 min on ice and blocked with 2% bovine serum albumin for 1 h at room temperature. Next, sections were incubated overnight at 4°C with the primary antibody (Proteintech, Chicago, USA). After three washes with PBS, sections were incubated with Alexa Fluor 488-conjugated secondary antibodies for 1 h and incubated in an area protected from light. The nuclei were stained with 4′,6-diamidino-2-phenylindole (DAPI). Finally, fluorescence images were acquired using confocal microscopy.

### In vivo tumor formation

Male BALB/C nude mice (6-week-old) were purchased from the Laboratory Animal Center at Xiamen University. Mice were randomly divided into three groups with 8 mice in each group. Each mouse in the shControl, shGIPC1, and rescue groups was subcutaneously injected with 100 μL of BGC-823 cells (10^7^ cells/mL) on a single side of the posterior flank. Seven days after the implantation of tumor cells, the tumor size was measured every 3 days. The formula used to calculate the tumor volume was as follows: V = 0.5 × D × d^2^ (V is the tumor volume, D is the longitudinal diameter, and d is the latitudinal diameter). When the tumor size became ~1,000 mm^3^, mice were euthanized using 5% isoflurane. The animal care procedures and animal experiments were ratified by the Laboratory Animal Ethics Committee (XMULAC20170297) of Xiamen University and complied with the Guidelines for the Care and Use of Laboratory Animals of the National Institutes of Health (NIH) and other relevant national regulations.

### Statistical analysis

Data of all assays were presented as the mean ± SEM values and analyzed using the Student’s *t*-test or one-way analysis of Variance (ANOVA) using GraphPad Prism software (Dotmatics, Boston, MA, USA). *p* < 0.05 was considered to indicate a statistically significant difference.

## Results

### GIPC1 expression is upregulated in GC

**Figure 1:**
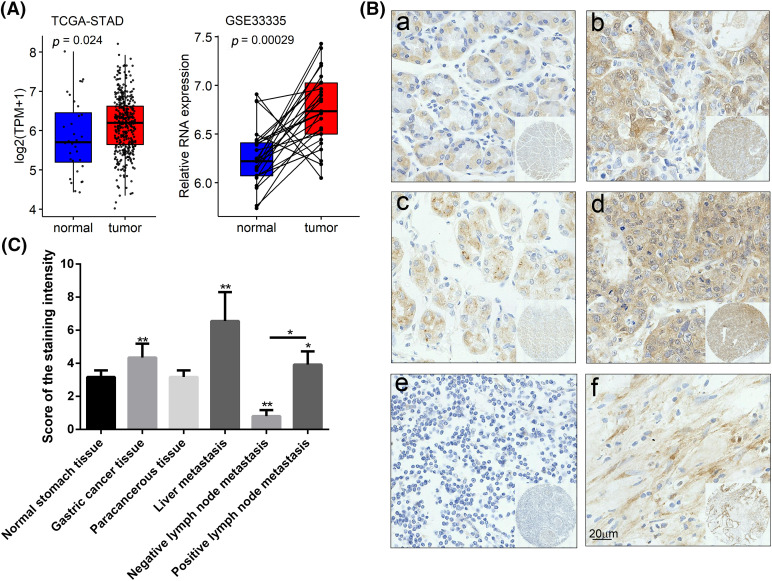
GIPC1 expression in GC. (A) A web server was accessed for analyzing the RNA sequencing expression data (Gene Expression Profiling Interactive Analysis), and GIPC1 expression in healthy individuals and cancer patients was compared with that in the gastric cancer dataset. (B) Representative immunohistochemistry images of GIPC1 expression in normal stomach tissues and different GC metastatic tissues. (a) Normal stomach tissue, (b) GC tissue, (c) paracancerous tissue, (d) liver metastasis, (e) negative lymph node metastasis and (f) positive lymph node metastasis. (C) GIPC1 expression in different GC tissues and normal stomach tissues. Statistical analysis was performed by one-way ANOVA. *p < 0.05, **p < 0.01.

GIPC1 expression was analyzed in GC tissues using TCGA. Upon comparing GIPC1 expression in healthy individuals (n = 32) and GC patients (n = 375), GIPC1 expression was found to be markedly higher in GC tissues than in normal gastric tissues (Fig. 1A). GIPC1 was also significantly upregulated in the tissue samples obtained from patients with GC compared to adjacent non-cancerous tissue samples (GSE38941). To further confirm the association between GIPC1 and GC development, GIPC1 protein levels were evaluated in GC samples on a tissue microarray in an immunohistochemistry assay. As shown in Figs. 1B, 1C, the expression levels of GIPC1 in tissues obtained from patients with GC and liver metastases were markedly higher than those in normal gastric tissues (*p* < 0.05). GIPC1 expression levels were also higher in patients with lymph node metastases than in those without lymph node metastases (Table 1). These results indicated that GIPC1 may be positively associated with GC development.

**Table 1:** Association between GIPC expression and clinicopathological factors in gastric cancer

Characteristic	N	GIPC1 immunohistochemical staining		*p*
		Low expression	High expression	
Age				
<60	38	36(94.7)	2(5.3%)	0.06
≥60	38	26(68.4%)	12(31.6%)	
Gender				
Male	59	48(81.4%)	11(18.6%)	1
Female	21	17(81.0%)	4(19.0%)	
Location				
Gastric antrum	24	18(75%)	6(25%)	0.529
Others	48	40(83.3%)	8(16.7%)	
T stage				
T1 + T2	11	10(90.9%)	1(9.1%)	0.408
T3 + T4	33	24(72.7%)	9(27.3%)	
N stage				
N(−)	20	11(100%)	0(0%)	0.049*
N(+)	40	29(72.5%)	11(27.5%)	
M stage				
M0	54	43(79.6%)	11(20.4%)	1
M1	18	14(77.8%)	4(22.2%)	
AJCC stage				
I + II	31	27(87.1%)	4(12.9%)	0.106
III + IV	34	24(70.6%)	10(29.4%)	

Note: *p < 0.05.

### GIPC1 promotes proliferation and migration in GC

**Figure 2:**
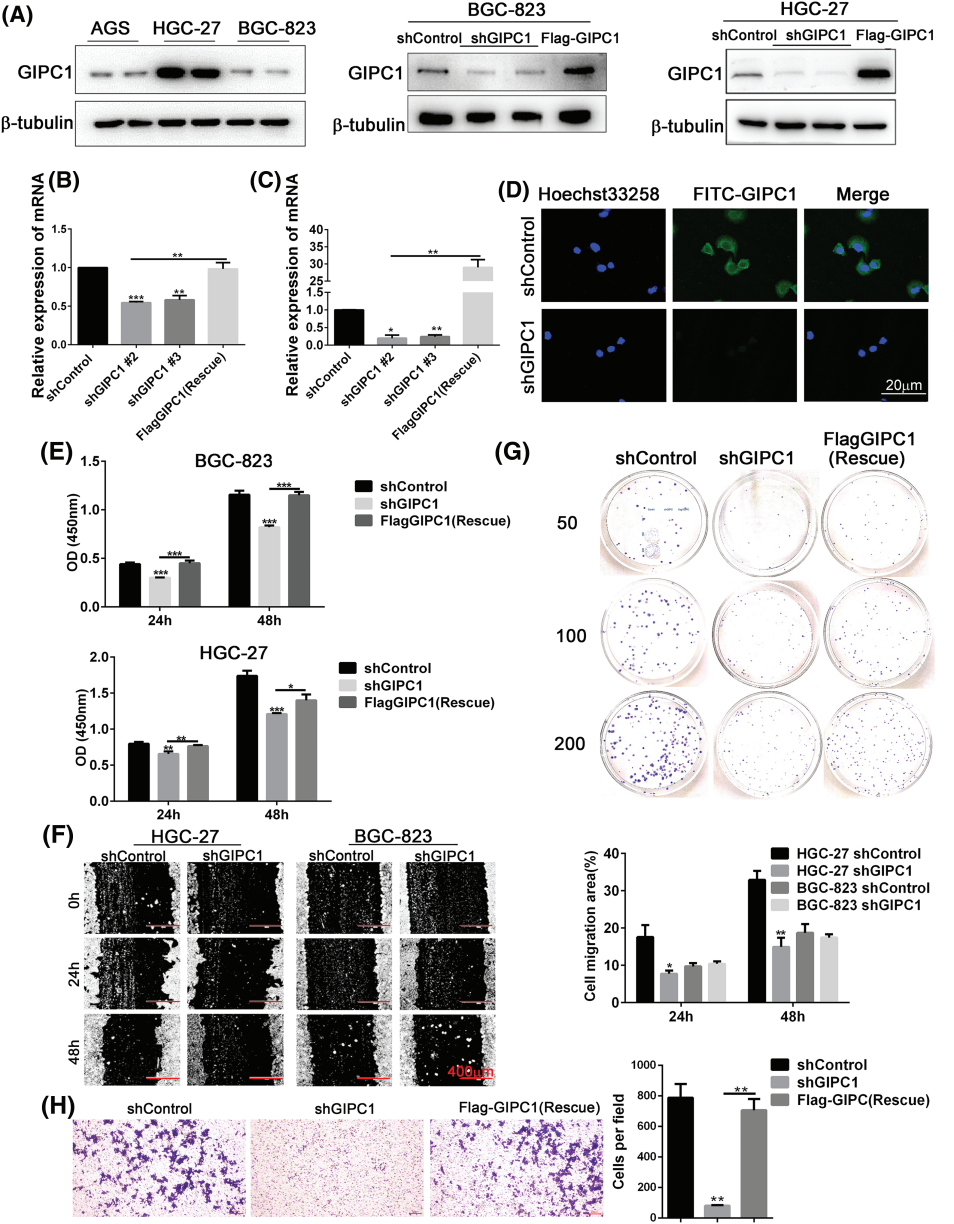
GIPC1 promotes proliferation, migration, and colony formation in GC cells. (A) GIPC1 expression in three GC cell lines, and GIPC1 protein expression in the BGC-823 and HGC-27 GC cell lines after GIPC1 knockdown and rescue. (B and C) GIPC1 mRNA expression in BGC-823 and HGC-27 GC cell lines after GIPC1 knockdown and rescue. (D) Immunofluorescence distribution in the BGC-823 GC cell line after GIPC1 knockdown. (E) Proliferation was examined in the BGC-823 and HGC-27 GC cells lines using a Cell Counting Kit-8 assay. (F) Migration of cells with GIPC1 knockdown was determined using a wound healing assay. (G) Colony formation assay in the HGC-27 GC cell line. (H) Invasion of cells exhibiting GIPC1 knockdown and rescue was identified using transwell assay. All data represent the mean ± SEM values from three independent experiments. Statistical analysis was performed by unpaired Student’s *t-*test. *p < 0.05, **p < 0.01, ***p < 0.001.

GIPC1 protein expression was detected in three GC cell lines (AGS, HGC-27 and BGC-823). As shown in Fig. 2A, the results of western blotting analyses revealed that GIPC1 protein levels of were higher in the HGC-27 GC cell line. Two GC cell lines, HGC-27 and BGC-823 were selected for use in subsequent experiments. First, BGC-823 and HGC-27 cell lines stably expressing GIPC1 shRNA were generated using lentiviral vectors. Protein and mRNA expression was verified (Figs. 2B, 2C). An immunofluorescence assay was performed to investigate GIPC1 distribution, and the results demonstrated that GIPC1 was mainly distributed in the cytoplasm, particularly close to the plasma membrane (Fig. 2D), which is consistent with the findings of a previous report [29].

CCK-8, colony formation, and wound healing assays were performed to detect the effects of GIPC1 expression on GC cell proliferation and migration. Compared to the shControl group, the proliferation rate of the knockdown group was markedly decreased, and the proliferation rate of the rescue group was markedly increased compared to that of the knockdown group (Fig. 2E). The results obtained for both GC cell lines were consistent at 24 and 48 h. These results suggest that GIPC1 could promote GC cell proliferation.

The migration rate of HGC-27 cells in the knockdown group was notably lower than that of cells in the shControl group (Fig. 2F), indicating that GIPC1 could promote the migration of HGC-27 cells. In addition, the results of transwell assays also found that GIPC1 could promote the invasion of HGC-27 cells (Fig. 2H). However, no notable migration of BGC-823 cells was observed at any timepoint. The colony formation assay demonstrated fewer colonies in HGC-27 cells transfected with shGIPC1 than in cells in the shControl and rescue groups (Fig. 2G). These results indicated that the silencing of GIPC1 effectively suppressed the ability of GC cells to proliferate, form colonies, and migrate.

### GIPC1 accelerates the proliferation and migration of GC via the PDGFR/PI3K/AKT signaling pathway

As an important tumor-promoting agent, PDGFR affects fibrogenesis [21] and regulates cell proliferation and differentiation via the PI3K/AKT signaling pathway [30,31]. In hepatic stellate cells, GIPC1 knockdown reduced the protein levels of p300, known to acetylate H3 at K27 [28]. H3K27ac reduced the extent of binding of the PDGFR-β promoter, while PDGFR-β mRNAs and proteins decreased and inhibited the extent of fibrogenesis. GIPC1 knockdown downregulated PDGFR-α protein levels via autophagic degradation to inhibit fibrogenesis [28]. Therefore, we hypothesized that GIPC1 might regulate the PI3K/AKT signaling pathway via PDGFR signaling, thereby affecting the proliferation and migration of GC cells.

**Figure 3:**
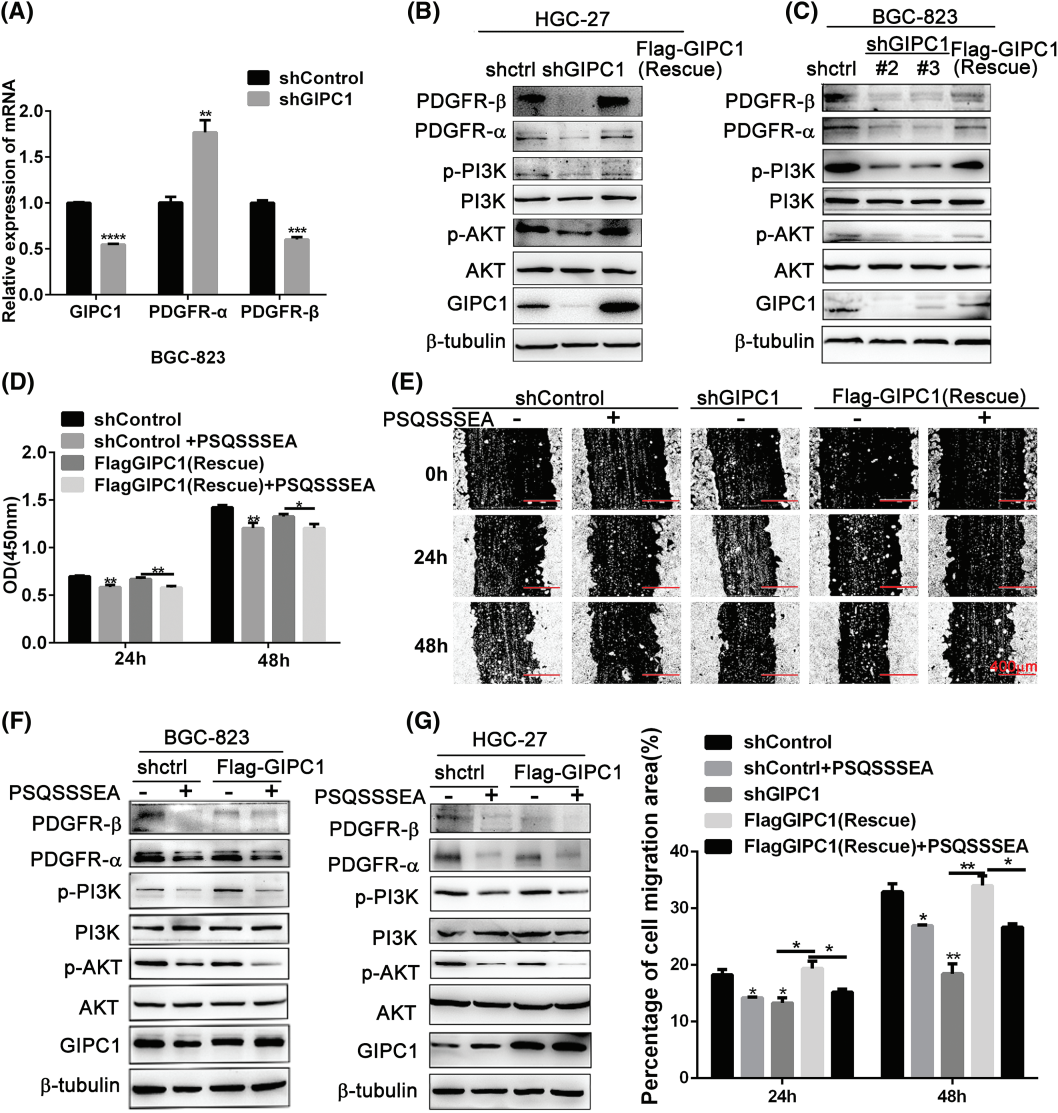
GIPC1 knockdown and GIPC blocking peptide reduce the expression of proteins involved in the PDGFR/PI3K/AKT axis and inhibit proliferation and migration in GC cells. (A) mRNA expression levels of PDGFR-α and PDGFR-β were examined via quantitative PCR. (B and C) Protein levels of PDGFR-α, PDGFR-β, p-PI3K, PI3K, p-AKT and AKT were examined via Western blotting. (D) GIPC blocking peptide suppressed the proliferation of BGC-823 GC cells. (E) GIPC blocking peptide suppressed the migration of HGC-27 GC cells in a wound healing assay. (F and G) Expression levels of PDGFR/PI3K/AKT-related proteins after the addition of GIPC blocking peptide to BGC-823 and HGC-27 GC cells. All data represent the mean ± SEM values from three independent experiments. Differences between groups were analyzed by Student’s *t-*test (A and G) or one-way ANOVA (E). *p < 0.05, **p < 0.01, ***p < 0.001, ****p < 0.0001.

In order to investigate this hypothesis, PDGFR and PI3K/AKT expression levels were detected in BGC-823 and HGC-27 cells after the knockdown and overexpression of GIPC1. GIPC1 knockdown reduced PDGFR-β mRNA levels in GC cells (Fig. 3A). The protein levels of PDGFR-α and PDGFR-β and phosphorylation levels of PI3K and AKT were markedly reduced after GIPC1 knockdown. Furthermore, GIPC1 overexpression compromised the adverse effects of GIPC1 silencing on PDGFR/PI3K/AKT protein expression (Figs. 3B, 3C). These results suggest that GIPC1 could upregulate PDGFR-α and PDGFR-β expression levels and activate the downstream PI3K/AKT cascade.

The GIPC1 blocking peptide (PSQSSSEA) was applied to confirm these results. The GIPC1 blocking peptide can competitively bind to the PDZ domain of GIPC1, and inhibit the functions of GIPC1. These results demonstrate that the proliferation and migration of GC cells in the shControl and rescue groups were significantly inhibited after GIPC blocking peptide addition (*p* < 0.01). The GIPC1 blocking peptide could markedly inhibit the proliferation and migration of GC cells (Figs. 3D, 3E).

Its molecular mechanism was assessed via western blotting (Figs. 3F, 3G). In both the shControl and rescue groups, PDGFR-α and PDGFR-β protein expression levels as well as PI3K and AKT phosphorylation levels were markedly reduced after treatment with the GIPC1 blocking peptide. The changes in the protein levels in the two GC cell lines (BGC-823 and HGC-27) were consistent. The results of this experiment were consistent with the effects of knockdown of GIPC1.

### PDGF-BB and AKT inhibitors affect the proliferation and migration in cells exhibiting the differential expression of GIPC1 in GC

PDGF-BB cytokines and AKT inhibitors were used to further demonstrate that GIPC1 promoted the proliferation and migration of GC cells via the mediation of the PDGFR/PI3K/AKT signaling pathway.

**Figure 4:**
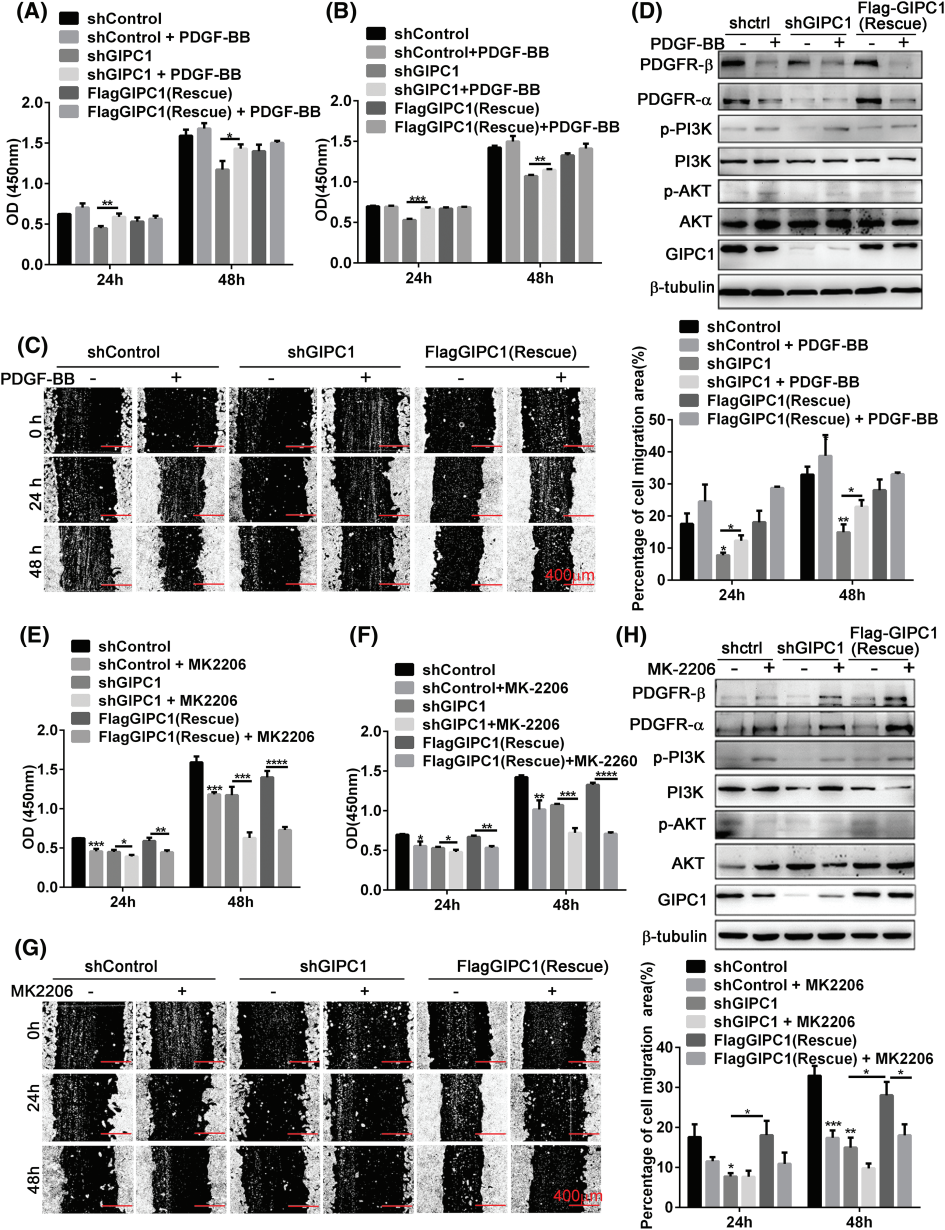
Effects of PDGF-BB and AKT inhibitor on the regulation of GC cell proliferation and migration by GIPC1 and its molecular mechanism. (A and B) PDGF-BB promoted the proliferation of BGC-823 and HGC-27 GC cells. (C) PDGF-BB facilitated the migration of HGC-27 GC cells in a wound healing assay. (D) Expression levels of PDGFR/PI3K/AKT-related proteins after the addition of PDGF-BB. (E and F) AKT inhibitor suppressed the proliferation of BGC-823 and HGC-27 GC cells. (G) AKT inhibitor decreased the migration of HGC-27 GC cells in a wound healing assay. (H) Expression levels of PDGFR/PI3K/AKT-related proteins after the addition of the AKT inhibitor. All data represent the mean ± SEM from three independent experiments. Differences between groups were analyzed by one-way ANOVA. *p < 0.05, **p < 0.01, ***p < 0.001, ****p < 0.0001.

PDGF-BB is a ligand of PDGFR. It can bind to PDGFR, activate PDGF-mediated signaling pathways, and exhibit corresponding biological functions. Proliferation was evaluated using CCK-8 assays, while migration was evaluated using wound healing assays. As demonstrated by the CCK-8 assay results, GIPC1 silencing inhibited cell proliferation compared to that observed in the shControl and rescue groups, while PDGF-BB addition increased cell proliferation. In particular, the growth rate in the knockdown group was more notable (Figs. 4A, 4B). As shown in Fig. 4C, PDGF-BB accelerated the rates of GC cell migration in each group. In particular, the knockdown group exhibited a marked increase in the migration of GC cells.

Since exogenous PDGF-BB increased the proliferation and migration of GC cells, it was verified whether the effects of exogenous PDGF-BB addition were associated with the PDGFR/PI3K/AKT signaling pathway. PI3K and AKT phosphorylation levels were markedly increased after PDGF-BB addition, especially the shGIPC1 group. Notably, PDGFR-α and PDGFR-β protein expression showed markedly reduced feedback induction, and GIPC1 expression levels were also slightly decreased in the shControl and rescue groups (Fig. 4D). Overall, PDGF-BB increased GC cells proliferation and migration, and it eliminated the suppressive effects induced by GIPC1 knockdown.

The proliferation and migration of BGC-823 and HGC-27 GC cells were markedly suppressed upon treatment with the AKT inhibitor MK-2206 (Figs. 4E–4H).

Thus, the present study explored the relationship between GIPC1 and the PDGFR/PI3K/AKT signaling pathway using western blotting techniques. The AKT phosphorylation was markedly decreased in the three groups upon treatment with the AKT inhibitor. Additionally, the PDGFR-α and PDGFR-β protein levels and PI3K phosphorylation levels were markedly increased (Fig. 4H). The experimental results further demonstrated that GIPC1 activated the downstream PI3K/AKT signaling pathway by upregulating PDGFR-α and PDGFR-β to promote GC cell proliferation and migration (Figs. 4E–4G).

### The silencing of GIPC1 suppresses tumor growth in a xenograft model

**Figure 5:**
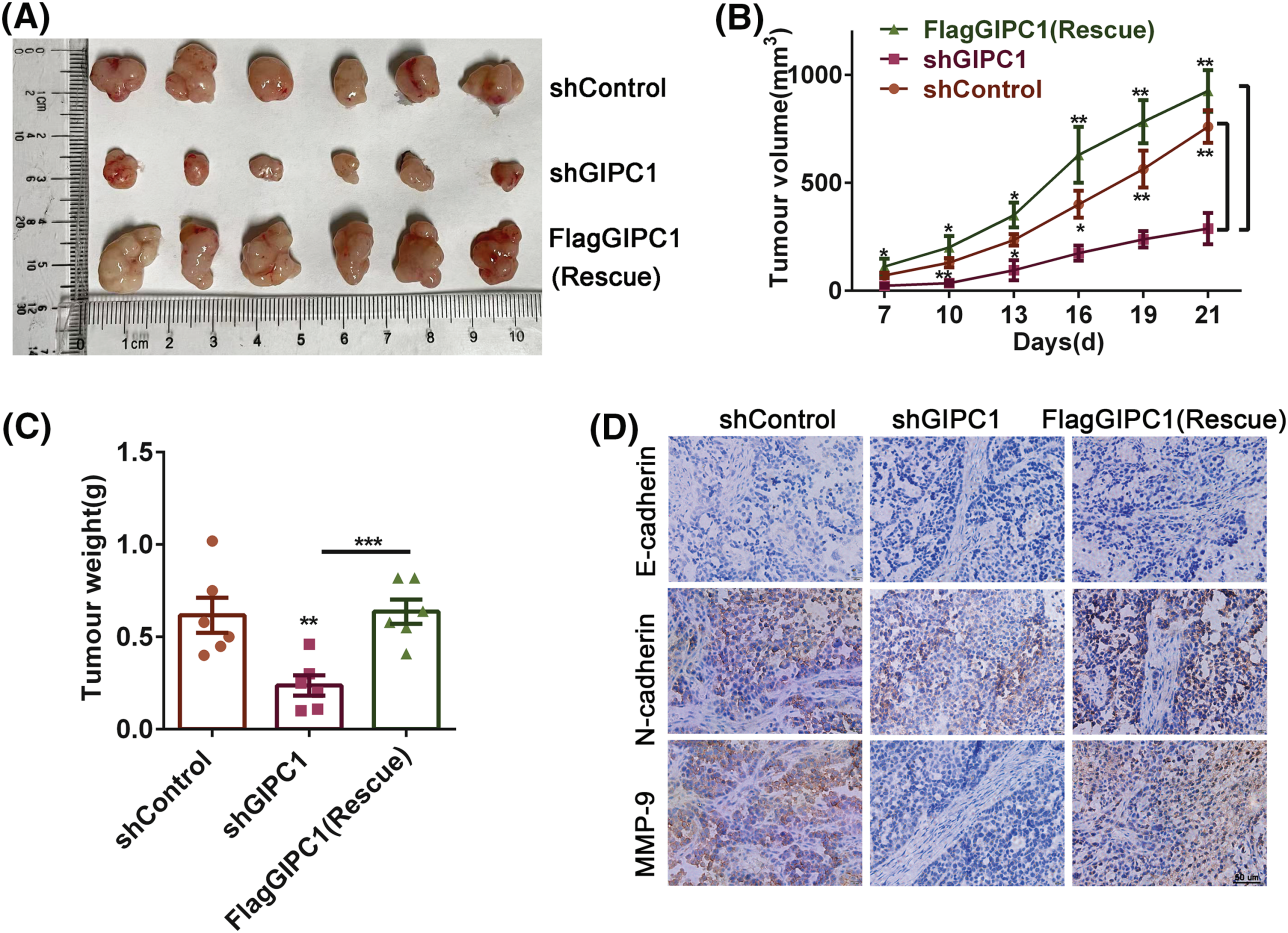
Knockdown of GIPC1 inhibits tumor growth and migration. (A) Mice were sacrificed 21 days after injection, tumors were collected, and images were captured. Tumor sizes were measured every 3 days. (B) Tumor volumes were compared among the shControl, shGIPC1 and rescue groups. (C) Tumor weights were compared among the shControl, shGIPC1, and rescue groups. (D) Tumor xenograft tissues were collected for immunohistochemical staining of E-cadherin, N-cadherin and MMP-9. Statistical analysis was analyzed by Student’s *t-*test. *p < 0.05, **p < 0.01, ***p < 0.001.

In order to determine the effect of GIPC1 on GC growth and metastasis *in vivo*, mice were randomly divided into three groups, i.e., the shControl, shGIPC1, and rescue groups. The results revealed that the volumes of GC xenograft tumors in the shGIPC1 group were markedly smaller than those in the shControl and rescue groups (Fig. 5A). GIPC1 knockdown could effectively inhibit tumor growth (Fig. 5B). In addition, the tumor weight in the shGIPC1 group was markedly reduced compared to that in the shControl and rescue groups (Fig. 5C). Subsequently, immunohistochemistry staining was used to determine the potential migratory ability of cells. N-cadherin and matrix metalloproteinase-9 (MMP9) expression levels were markedly reduced in all tumor tissues in the shGIPC1 group, as compared to those in the shControl and rescue groups (Fig. 5D). Taken together, our findings were in accordance with those of *in vitro* experimental studies and showed that GIPC1 silencing markedly inhibited the proliferation and migration of GC cells.

## Discussion

GIPC1 belongs to the GIPC protein family, and its PDZ domain can bind to various protein molecules. It can interact with IGF-1R [32] and TGFβ-IIIR [33] to regulate the proliferation of breast cancer cells. The PDZ domain of GIPC1 promotes angiogenesis by binding to the SEA domain of NRP1 [34]. GIPC1 is a key protein involved in protein trafficking, endocytosis, receptor aggregation, and tumor occurrence and development [35]. However, to our knowledge, the role of GIPC1 in GC has not been investigated previously. Our findings demonstrate that GIPC1 expression levels were higher in GC and liver metastasis tissues than in normal gastric tissues. GIPC1 expression levels were associated with lymph node metastasis. Evidence has indicated that GIPC1 is positively associated with GC occurrence and metastasis to a certain extent, suggesting that GIPC1 expression levels could potentially be used to screen indicator for GC.

In breast cancer, GIPC1 can facilitate cell proliferation, survival, and invasion via signaling pathways such as the AKT/Mdm2/p53 and insulin-like growth factor 1-induced extracellular signal-regulated kinase (ERK)1/2 signaling pathways [36,37]. In pancreatic cancer, the PDZ domain of GIPC1 can stabilize IGF-1R proteins and contribute to cell proliferation [12]. Our findings helped to identify that GIPC1 could promote the proliferation and migration of GC cells. GIPC1 knockdown resulted in a consistent decrease in protein and mRNA levels of PDGFR-β. A previous study has demonstrated that GIPC1 forms a complex with myosin VI to transport vesicles [7] that could contain p300. GIPC1 downregulation prevented nuclear localization, weakened the p300 activity, and downregulated the H3K27ac promoter of PDGFR-β, thereby downregulating of PDGFR-β mRNA transcription [28]. GIPC1 and the autophagy molecule Myb1, a membrane transporter, have the same binding site as myosin VI, and GIPC1 can regulate the autophagy of specific proteins [38]. GIPC1 and RAB GTPase-dependent endosomal targeting protects PDGFR-α from autophagic degradation. After GIPC1 knockdown, PDGFR-α protein levels were decreased with an increase autophagic degradation rather than through transcriptional regulation [28]. PDGF binds to PDGFRs and activates downstream signaling pathways. Dimerized and activated PDGFRs can interact with SH2 domain-containing signaling proteins, activating several signaling pathways, such as the MAP kinase pathways, and PI3K/AKT and phospholipase Cγ pathways [39]. Thus, GIPC1 may upregulate PDGFR-α and PDGFR-β and then activate the downstream PI3K/AKT signaling pathway to promote the proliferation and migration of GC cells.

The GIPC blocking peptide is an artificially designed and synthesized peptide based on the PDZ domain of GIPC1. It can efficiently and competitively bind to the PDZ domain and block the signaling pathway downstream of GIPC1 [40]. After the supplementation of GC cells in the shControl and rescue groups with GIPC blocking peptides, the protein levels of PDGFR-α, PDGFR-β, p-PI3K and p-AKT were all downregulated, which inhibited PDGFR/PI3K/AKT signaling and cell proliferation and migration. This was consistent with the results observed after the knockdown of GIPC1.

PDGF-BB, a ligand for PDGFR, is a polypeptide growth factor produced by platelets and vascular endothelial cells. While interacting with PDGFR, it can promote cell division and proliferation [41]. After treating GC cells with PDGF-BB, PDGF-BB and PDGFR combine with each other, resulting in PDGFR being subjected to various complex regulatory processes. Some PDGFRs undergo lysosomal degradation, while some PDGFRs undergo autophosphorylation, resulting in PDGFR downregulation [42,43]. However, PDGFR/PI3K/AKT signal transduction was enhanced, facilitating the proliferation and migration of GC cells, and weakening GIPC1 knockdown-induced effects.

MK-2206 is a highly selective inhibitor of AKT1/2/3 that can suppress the phosphorylation of AKT at threonine 308 and serine 473, thereby blocking the AKT signaling pathway. AKT inhibitors inhibit AKT phosphorylation prevent downstream molecule signaling, and impair cell proliferation and migration. AKT inhibitors suppress AKT phosphorylation and block AKT downstream signaling pathways, while upstream signaling molecules such as PDGFR-α, PDGFR-β, and phosphorylated (p-)PI3K increase feedback induction.

## Conclusion

In summary, the present study investigated the expression and potential functions of GIPC1 in GC. GIPC1 was upregulated in tissues obtained from patients with GC and liver metastasis. GIPC1 knockdown decreased the PDGFR-α and PDGFR-β expression levels inhibited the PI3K/AKT signaling pathway, and subsequently led to impeded the proliferation and migration of GC cells. These findings suggest that GIPC1 is essential for regulating cell proliferation and migration via the PDGFR/PI3K/AKT signaling pathway.

## Data Availability

All data and materials have been made available.
